# Neonatal unit (NU) Infant Medical Trauma and the lifelong implications for children and their families

**DOI:** 10.3389/fpsyg.2026.1803035

**Published:** 2026-08-27

**Authors:** Rupa Rubinstein, Narendra Aladangady

**Affiliations:** 1 Homerton Hospital, London, United Kingdom; 2 Blizard Institute, Queen Mary University of London, London, United Kingdom

**Keywords:** Adverse Childhood Event (ACE), Family Integrated Care (FICare), Infant Medical Trauma, neonatal unit, Post-Intensive Care Syndrome (PICS), trauma-informed care

## Abstract

**Introduction:**

Infant Medical Trauma (IMT) is a result of prolonged toxic experiences usually experienced by patients in the neonatal unit (NU), and is associated with significant sequalae.

**Method:**

This article describes the pathophysiology of IMT and the short- and long-term impacts. The possible methods of minimising this is discussed.

**Results:**

Increased neonatal staff and parent awareness of IMT could encourage clustering of care, improved use of tools for recognition of pain and further highlight the benefits of family-integrated care and skin-to-skin care.

**Conclusion:**

While IMT may be considered a consequence of survival from the NU, it’s effects can be minimised with a range of techniques to protect the patients (and parents) from psychological harm.

## Introduction

Worldwide, approximately 1 in 10 babies are born unwell or premature (less than 37 weeks gestation compared to full term of 40 weeks) (World Health Organization, 2023). Survival to discharge rates are increasing even for the most premature babies born less than 26 weeks gestation (Varsami et al., 2025). Attention is now moving to quality of life and psychological outcomes in childhood and beyond.

Infant Medical Trauma is a type of Adverse Childhood Event (ACE) that occurs in unwell babies or premature infants. It usually refers to the experiences of young, often premature infants within the intensive care setting such as the neonatal intensive care unit (NU). This is separate to the medical events such as sepsis or intraventricular haemorrhage that may also impact neurodevelopment.

Adults who were born prematurely are known to have increased rates of autism, attention deficit hyperactivity disorder (ADHD; Johnson and Marlow, 2011; Allen et al., 2020), psychiatric diagnoses (Johnson and Marlow, 2011; Anderson et al., 2021), and they form fewer romantic relationships (Mendonça et al., 2019).

The very fact of being born prematurely may disrupt normal maturation of the brain and predispose the premature neonate to these conditions, however, other factors should also be considered such as the noxious environment and lack of consistent presence of a safe familiar primary caregiver.

## Theory and clinical context

The concept of Infant Medical Trauma (IMT) was described by D’Agata et al. (2016). The term encapsulates the prolonged negative experiences of an (usually premature) infant in the neonatal unit. Instead of the dark, warm, safe and constrained environment of the womb, the neonate experiences repeated noxious stimuli. Some may be obvious such as intubations, nasogastric tube insertions (tubes inserted from the nose to the stomach for feeding), intravenous cannulations and other invasive tests and investigations. More subtle adverse stimuli include the bright lights, noisy environments, temperature variations, distressing sensations such as nappy changes or offensive smells, e.g., alcohol gel. Unpleasant tastes and oral sensations, and repeated physical examinations usually at the convenience of the healthcare provider add to the concept of IMT. In addition to this, a baby who experiences a period of acute illness will have increased clinical examinations and investigations, parental separation and sleep deprivation or fragmentation. As is the nature of intensive care, these frequent clinical examinations and interventions are often necessary to preserve the life of the patient.

The American Academy of Pediatrics’ (AAP) technical report (2011) refers to this “severe, frequent and/or prolonged stress” as toxic and that this may disrupt the child’s physiology and result in long term health effects (Shonkoff et al., 2012). This level of toxic stress is unable to be adequately buffered or ameliorated by positive socio-emotional support (Weber and Harrison, 2019). In a traditional neonatal setting, the patient often endures frequent toxic cumulative (life-saving) events alone, without support or comfort from their parents or caregivers. Simons’ prospective study noted an average of 14 painful procedures per day in the first 2 weeks of life (Simons et al., 2003).

## Pathophysiology

It is hypothesised that repeated or persistent toxic stress overwhelms the premature infant causing prolonged activation of their body’s stress response system. Without any mechanisms to buffer this, chronic inflammation activates the hypothalamic-pituitary axis (Mooney-Leber and Brummelte, 2017) leading to a pro-inflammatory state and altered sympathetic stress reactivity. This results in changes in brain volume, white matter injury, neuronal inflammation, maladaptive neuronal pruning and alterations in the brain’s microstructure (Vinall and Grunau, 2014; D’Agata et al., 2016).

**FIGURE 1:**
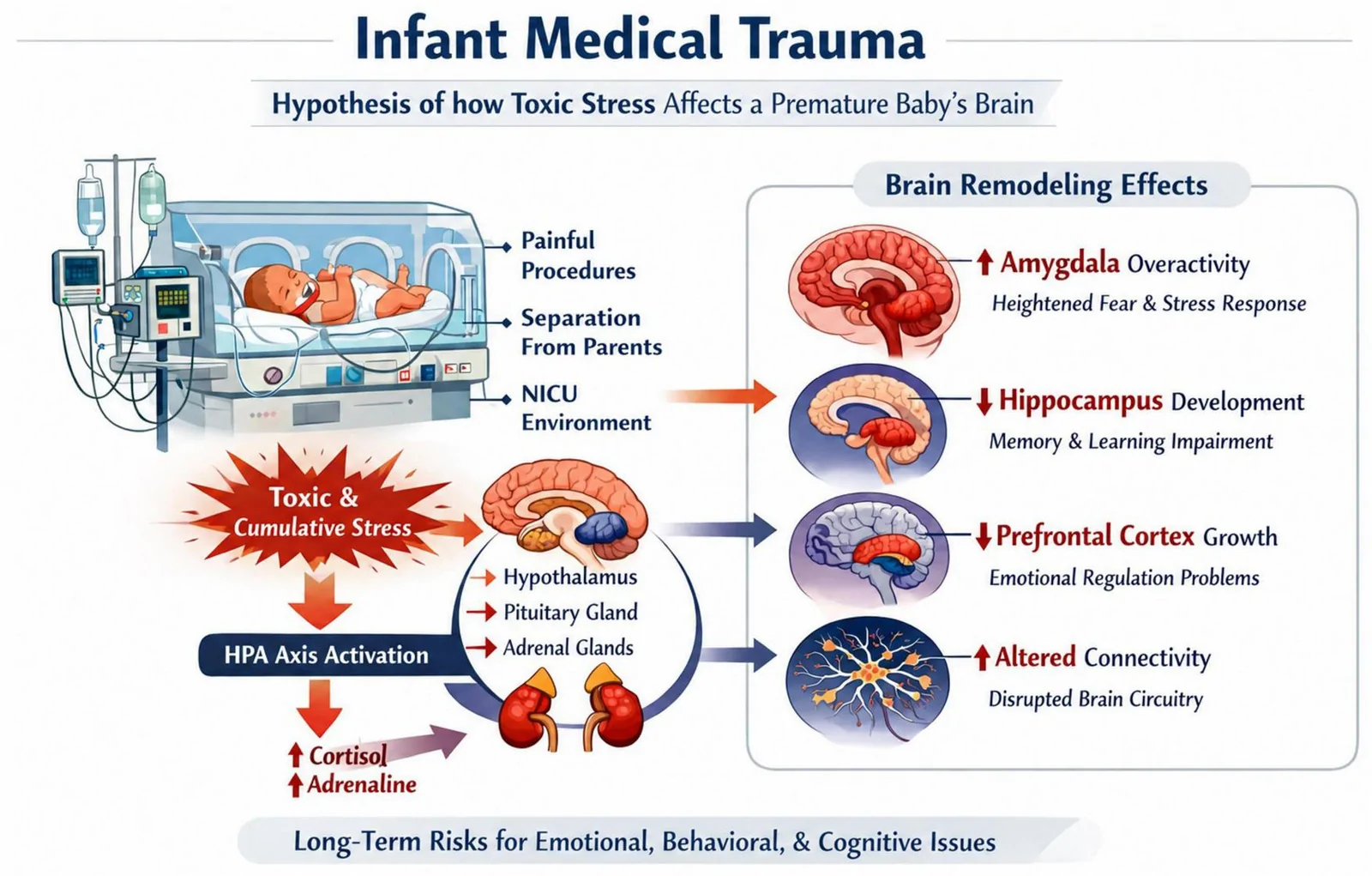
Hypothesised mechanism of how toxic stress affects the premature baby’s brain.

This modelling of the neurological pathways in the brain in response to persistent toxic stress (Figure 1) can lead to prolonged maladaptive responses to stressors into adulthood (Mooney-Leber and Brummelte, 2017). This is associated with poorer long-term psychological outcomes such as increased risks of social difficulties, attention challenges and adverse mental health symptoms (Räikkönen et al., 2008; Grunau, 2013; Berens et al., 2017). Malin et al. (2023) reported evidence of epigenetic changes responsible for the regulation of serotonin, dopamine and cortisol. The degree of methylation correlates with the degree of adverse symptoms reported by the ex-premature NU patients (Malin et al., 2023).

## Short term impacts in the NU

Gut dysbiosis, chronic inflammation and metabolic dysregulation are seen in the shorter term (D’Agata et al., 2016; Coughlin, 2017). Malin et al.’s (2023) scoping review summarised the short term impacts as suboptimal reflexes, less sustained attention, slow inhibited approach, poor self-regulation, poor quality movement and a baby who is noted to be highly aroused and excitable (Malin et al., 2023). These signs are highly prognostic for dysfunctional and pathological neurodevelopment particularly during the period which would have been the third trimester of pregnancy.

## Long term impacts

### School age

There are many long-lasting sequelae for NU graduates and starting school can be an anxious time for parents as they navigate adapting to school life. The new challenges and demands of being a student may also reveal previously unidentified social and academic difficulties.

Children who have experienced altered modelling of their neural pathways are more easily overwhelmed and struggle to sustain attention or self-soothe; this elicits different care-giver responses. A child who experiences sensory overstimulation can struggle to maintain focus on their learning in a school environment.

Anderson’s meta-analysis of 10 studies including individual patient level data of over 3000 premature and term controls showed that children born prematurely were at increased risk of ADHD, anxiety and mood disorders (Anderson et al., 2021).

Wolke et al. (2015) reported that across the German Bavarian Longitudinal Study and the UK EPICure Study, preterm children were more likely to be bullied at primary school age (ages 6/7 and 10/11) compared to their term peers. This result related to raised emotional problem scores despite controlling for disability, sex, socio-economic status and pre-existing emotional problems (Wolke et al., 2015). This suggests a further factor may increase the vulnerability of the preterm cohort.

A further study of 101 7-year-olds born ≤32 weeks gestation showed that increased parental stress and painful (skin-breaking) procedures in NU were associated with higher internalising behaviours in children who were not ventilated. Children who were ventilated and exposed to morphine had higher internalising behaviours compared to those not exposed to morphine (Ranger et al., 2014).

Ranger et al.’s (2015) neuroimaging of 56 seven year olds born less than 32 weeks gestation also showed that very preterm children exposed to painful procedures had smaller cerebellar volumes related to motor-visual integration and related to poor cognitive capabilities.

Perception of pain remains altered at 7.5 years of age as seen in Valeri et al.’s (2016) prospective study of 56 premature children born less than 32 weeks gestation. The children who had experienced a higher number of invasive procedures in the neonatal unit (and whose parent’s scored higher for Trait Anxiety) reported increased pain intensity (Valeri et al., 2016).

### Adolescence to adulthood

There is increasing evidence of the long-term impacts of prematurity on physical health of adults in middle age. Crump et al. (2019) conducted a cohort study of 4.3 million singleton births in Sweden and showed that by the age of 45, lower gestational age at birth was associated with increased all-cause mortality compared to term babies- 172.79 per 100,000 compared to 33.45 per 100,000. The effect was more pronounced in males compared to females and was seen for psychiatric as well as physical diagnoses (Crump et al., 2019).

Healy et al.’s (2013) case-controlled cohort study demonstrated that very preterm (≤32 weeks gestation) adolescents had socialisation problems in mid-adolescence which was associated with volumetric changes in the “emotion-processing brain network” when compared to term controls. They highlighted that this “atypical social development” increased the ex-very preterm adolescents’ vulnerability to psychiatric disorders (Healy et al., 2013). This finding follows a similar path to Kajantie et al.’s (2008) Swedish cohort study of 162 VLBW adults who found that they took longer to achieve normal “adult milestones” such as leaving home, gaining employment and even having children when compared to term controls. This suggests a lifetime of “catching up” with their peers (Kajantie et al., 2008).

Mendonça et al., 2019 systematic review and meta-analysis included 21 prospective longitudinal studies encompassing 4,423,789 adult participants. Preterm or low-birth weight adults were less like to have experienced a romantic relationship, have had sexual intercourse or become parents. There was a dose-response effect seen with respect to prematurity, and parenthood or romantic relationships. Quality of romantic relationships and peer social support were not impacted (Mendonça et al., 2019).

Ni et al. (2021) conducted a meta-analysis of individual patient level data across 5 cohort studies encompassing 2 international consortia. This assessed self-perceived social functioning in adults born ≤32 weeks gestation or <1500 g compared to term controls. The very premature or low birthweight adults perceived their relationships with friends to be significantly lower than their term peers suggesting poorer social support. Reassuringly, relationships with family and partners, as well as work and schooling experiences did not show a significant difference (Ni et al., 2021).

## Reducing the impact of IMT

Although difficult to disentangle from adverse physical events, it is clear that there is a significant sustained psychological impact to patients who requires lengthy NU hospitalisation. Post-Intensive Care Syndrome (PICS) is a well-recognised phenomenon in adult medical practice (Faculty of Intensive Care Medicine, 2025). This is relevant for the healthcare workers who may have contact with the child from birth through to adulthood.

Mooney-Leber and Brummelte (2017) and D’Agata et al. (2016) both highlight that a “Family Integrated Care” (FICare) approach could help ameliorate the impact of IMT. Schore’s regulation theory proposes that structural and functional changes in the premature infant’s brain can be induced by positive maternal-infant interactions (Schore, 2001). This means that the neurodevelopment of these fragile infants could be positively altered through the presence of a safe and loving caregiver.

An RCT of premature babies exposed to a FICare approach or not showed that a “family nurture intervention” had significant changes in EEG recordings at term suggesting improved neuro-behavioural outcomes seen at term corrected age (Welch et al., 2014).

Identification of pain may be challenging in the NU but various pain identification models exist such as N-PASS (Hummel et al., 2008) or the FLACC scoring system (Merkel et al., 1997). Pain scoring forms a routine part of neonatal nursing (and medical care) with recommendations for pharmacological and non-pharmacological pain management included. Non-pharmacological methods such as swaddling, non-nutritive sucking (use of a dummy or pacifier) (Akbari et al., 2022) and holding a baby may reduce the impact of uncomfortable or painful experiences (Campbell-Yeo et al., 2019). Johnston et al.’s (2017) Cochrane Review concluded that skin-to-skin care conferred an analgesic effect for procedural pain in neonates. Additionally, Campbell-Yeo et al.’s (2019) single blinded RCT showed that skin-to-skin care (with or without 24% sucrose) had a sustained analgesic effect for repeated painful procedures for preterm infants in the NU.

Feldman et al.’s (2014) Israeli cohort study showed that premature babies who received more skin-to-skin care had more organised sleep-wake cycles at term and better autonomic functioning. They had a less heightened stress response and were able to recover from stress more quickly compared to peers who did not receive skin to skin. Feldman also noted greater mother-child reciprocity persisted to 10 years of age which may also have protective effects on child mental health following IMT (Feldman et al., 2014).

Latva’s retrospective Finnish study from 1989 linked infrequent parental visits to the NU with increased behavioural and emotional problems in childhood. The risk was greatest if the mother’s visits were reduced and were a stronger risk factor “for later psychological development than the medical risks of the preterm infant” (Latva et al., 2004). This may be explained by the reduction in protective effects of the parental visits and unmitigated IMT experienced by the vulnerable NU patient.

With multi-disciplinary teamwork, procedures such “routine” clinical examinations, nappy changes and suctioning of medical devices could be clustered to allow the baby prolonged uninterrupted rest or skin-to-skin care, and to minimise physiological instability. Although, clustered care may cause prolonged stress without recovery. Therefore, minimising unnecessary handling and an individualised approach is optimal (BAPM, 2025). Staff and parent education on how to gently rouse and prepare babies for procedures and how to identify and respond their emotional state may also assist with maintaining a lower stress response. These evidence-based strategies reduce short-term stress and pain, and thus the cumulative effect may be of reducing the IMT experienced by the infant.

## Pharmacological pain relief

Historically it was believed that newborns did not experience pain (Anand and Hickey, 1987) and therefore analgesia or sedation was not prescribed. There is evidence to suggest that commonly used analgesia such as morphine infusions are associated with neuronal apoptosis (Hu et al., 2002) making some clinicians hesitant to use them. This disregards the short- and long-term impacts of repeated exposure to pain compared to the ill effects of the drugs. Prudent use of pharmacological analgesia is required to balance risks and benefits.

## Trauma informed care and Family Integrated Care (FICare)

**FIGURE 2:**
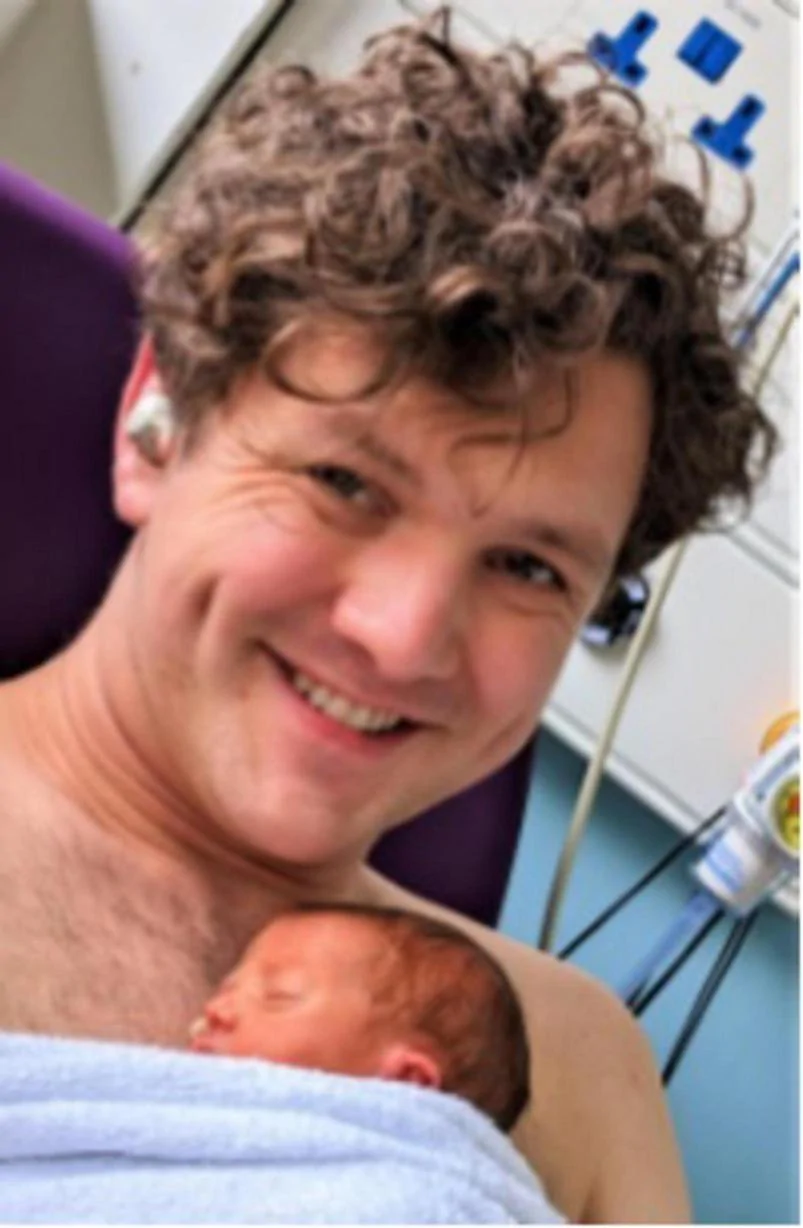
Parent performing skin-to-skin care with their premature infant.

FICare is a culture in the neonatal unit whereby parents or other primary caregivers, are considered to be partners in care for their hospitalised infant, and their prolonged presence is expected on the neonatal unit (Franck and O’Brien, 2019; BAPM, 2021). Parents are not considered to be visitors, simply partners in care. A key aspect of FICare is the presence and positive touch of the caregivers. This is exemplified in skin-to-skin care (Figure 2) which has short and longer term impacts for both baby and parents (Levin, 1994; Feldman et al., 2014; Franck and O’Brien, 2019; Pados and Hess, 2020).

Skin-to-skin care could be considered as trauma-informed care. Non-pharmacological methods of pain relief can be performed by any caregiver if the baby is clinically stable (Koukou et al., 2022). The FICare approach empowers parents with the knowledge, skills and confidence to care for their baby and provide non-pharmacological support during painful procedures and may thus optimise outcomes for the baby (BAPM, 2021).

## Dyadic trauma

Although this article focuses on IMT, it would be remiss not to consider the trauma faced by parents of babies in the NU. Stress, anxiety, depression and Post-Traumatic Stress Disorder (PTSD) symptoms are widely reported in mothers and fathers of hospitalised neonates (Kong et al., 2013; Koliouli et al., 2016; O’Brien et al., 2018; Rubinstein, 2024). The birth of a preterm or sick infant could be unexpected but may follow a high-risk pregnancy where both parents who were already grieving the loss of a healthy pregnancy are then faced with a traumatic birth followed by separation from their sick infant. Poor physical health may impact the maternal presence in the early days of admission further distancing the infant from their primary caregiver. Uncertainty over survival of the critically unwell infant, fear for the future and distress at seeing their baby so medicalised could inhibit parental presence and thus their support for their infant. Practical and financial challenges to being on the NU should not be underestimated for stressed and overwhelmed parents (BLISS, 2022).

In the UK, psychological support for parents is becoming an expected standard of care (BAPM, 2021). By supporting parents through their own trauma, they are then more able to be present to support their infant through their hospitalisation.

## Implications for neonatal policy developers and practitioners

The architectural design of the NUs is an essential part of delivering trauma-informed care. This, combined with specialised service delivery can welcome and provide space for families and other supportive allied-healthcare professionals. In the United Kingdom, this is becoming an expected standard of care (BAPM, 2021). This could include;

- Unrestricted parental presence with adequate facilities for parents to rest, eat and sleep near their baby’s cotside or closer to the NU.
- Psychological support for parents
- Training in trauma-informed care for all staff on the NU
- Parent education on how to support their baby neurodevelopmentally and psychologically during their hospitalisation.

## Implications for those involved in the care for ex-NU patients and their families

All professionals involved in the care of ex-premature or NU babies must be cognisant of the child’s medical history, especially as survival rates to discharge continue to improve. This includes primary and secondary school teachers, general practitioners, adult medical staff and the therapists and counsellors who may be involved in supporting these patients.

## Conclusion

As medical care advances in the NU, there is an growing risk of IMT as babies experience increasingly complex and intensive care and survive to adulthood. Quality of psychological health of the ex-NU patients can be improved through robust pain assessment and a FICare trauma-informed culture in the NUs to support parents psychologically so that parents are better equipped to support their baby in the NU.
